# Stratified primary care versus non-stratified care for musculoskeletal pain: qualitative findings from the STarT MSK feasibility and pilot cluster randomized controlled trial

**DOI:** 10.1186/s12875-020-1098-1

**Published:** 2020-02-11

**Authors:** Benjamin Saunders, Jonathan C. Hill, Nadine E. Foster, Vince Cooper, Joanne Protheroe, Adrian Chudyk, Carolyn Chew-Graham, Bernadette Bartlam

**Affiliations:** 1 Primary Care Centre Versus Arthritis, School for Primary, Community and Social Care, Keele University, Keele, Staffordshire ST5 5BG UK; 2 Keele Clinical Trials Unit (CTU), School for Primary, Community and Social Care, Keele University, Keele, Staffordshire ST5 5BG UK; 3 Family Medicine and Primary Care, Lee Kong Chian School of Medicine, Nanyang Technological University, Singapore, 308232 Singapore

**Keywords:** Musculoskeletal pain, Stratified care, Prognostic risk, Primary care, General practice, Qualitative

## Abstract

**Background:**

Stratified care involves subgrouping patients based on key characteristics, e.g. prognostic risk, and matching these subgroups to appropriate early treatment options. The STarT MSK feasibility and pilot cluster randomised controlled trial (RCT) examined the feasibility of a future main trial and of delivering prognostic stratified primary care for patients with musculoskeletal pain. The pilot RCT was conducted in 8 UK general practices (4 stratified care; 4 usual care) with 524 patients. GPs in stratified care practices were asked to use i) the Keele STarT MSK development tool for risk-stratification and ii) matched treatment options for patients at low-, medium- and high-risk of persistent pain. This paper reports on a nested qualitative study exploring the feasibility of delivering stratified care ahead of the main trial.

**Methods:**

‘Stimulated-recall’ interviews were conducted with patients and GPs in the stratified care arm (*n* = 10 patients; 10 GPs), prompted by consultation recordings. Data were analysed thematically and mapped onto the COM-B behaviour change model; exploring the Capability, Opportunity and Motivation GPs and patients had to engage with stratified care.

**Results:**

Patients reported positive views that stratified care enabled a more ‘structured’ consultation, and felt tool items were useful in making GPs aware of patients’ worries and concerns. However, the closed nature of the tool’s items was seen as a barrier to opening up discussion. GPs identified difficulties integrating the tool within consultations (Opportunity), but found this easier as it became more familiar. Whilst both groups felt the tool had added value, they identified ‘cumbersome’ items which made it more difficult to use (Capability). Most GPs reported that the matched treatment options aided their clinical decision-making (Motivation), but identified some options that were not available to them (e.g. pain management clinics), and other options that were not included in the matched treatments but which were felt appropriate for some patients (e.g. consider imaging).

**Conclusion:**

This nested qualitative study, using the COM-B model, identified amendments required for the main trial including changes to the Keele STarT MSK tool and matched treatment options, targeting the COM-B model constructs, and these have been implemented in the current main trial.

**Trial registration:**

ISRCTN 15366334.

## Background

Musculoskeletal (MSK) pain is common and associated with significant impacts for the individual and society [1]. Many MSK problems are managed predominantly in primary care [2], and in the UK, these account for 14% of general practice (GP) consultations [3, 4]. Usual primary care for people with MSK pain commonly follows a ‘stepped, wait and see’ approach, with patients initially given low intensity and low cost treatments, moving onto higher intensity or costlier treatments if needed [5]. An alternative approach is to stratify patient care according to the patient’s risk of poor outcome, e.g. persistent disabling pain. A model of stratified primary care, known as STarT Back, has been shown in the UK to be clinically- and cost-effective for non-specific low back pain (LBP) [6–8]. This approach involves the use of a brief stratification tool to identify patients’ risk of persistent disabling pain (low, medium or high) [9], and then matching risk subgroups to treatments. Using this approach, patients who need more intensive treatment are identified early, allowing them to be ‘fast-tracked’ to that treatment, whilst patients at low risk can be reassured of their good prognosis and avoid unnecessary treatments. Recent guidelines in the UK and elsewhere [10, 11] now recommend prognostic stratification for low back pain in primary care.

**Fig. 1:**
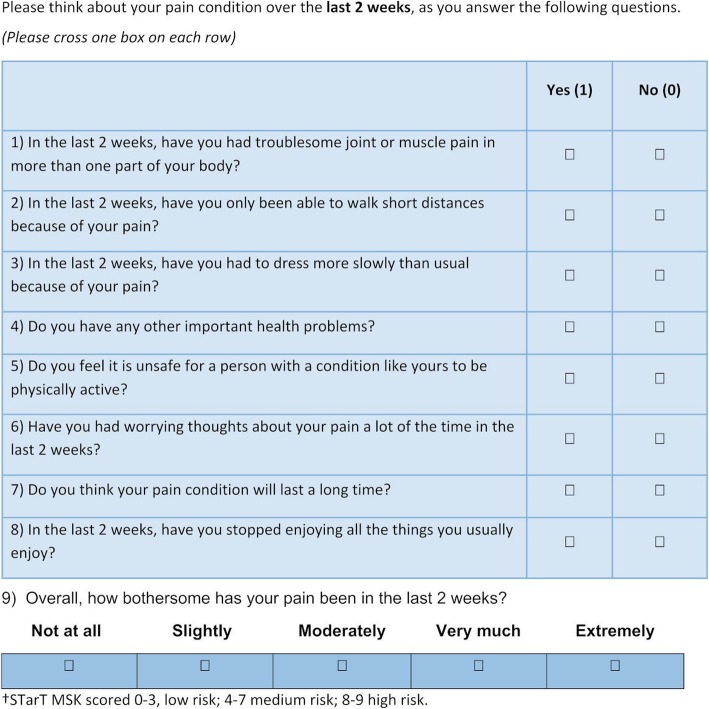
Development version of the Keele STarT MSK Tool© Keele University

**Fig. 2:**
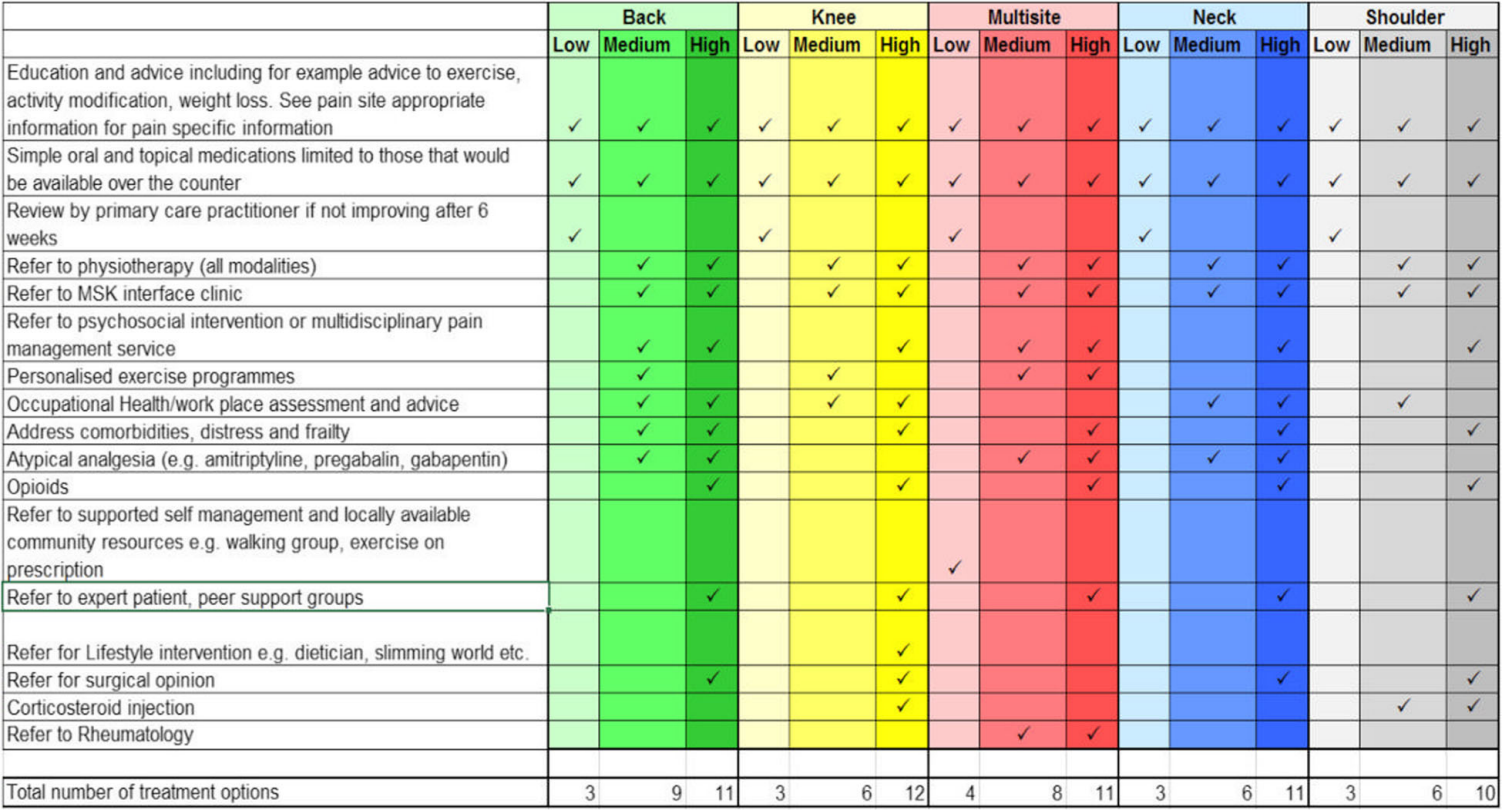
Recommended matched treatment options used in the STarT MSK feasibility and pilot RCT

Recent systematic reviews have evidenced the similarity in factors predicting outcome irrespective of different MSK pain sites [12, 13], and previous analysis of a modified version of the STarT Back tool showed it can predict outcome similarly across patients with back, neck, upper limb, lower limb or multisite pain [14], but needed refinement. Therefore, a development version of the Keele STarT MSK tool was produced to stratify primary care MSK pain patients into low, medium or high risk subgroups based on their risk of persistent disabling pain (see Fig. 1 below) and, through evidence reviews [15] and consensus group research [16], matched treatment options were identified (see Fig. 2, below). Prior to a main trial that compares the clinical- and cost-effectiveness of stratified primary care for patients with the five most common MSK pain presentations, versus usual, non-stratified care, a feasibility and pilot RCT was needed to test the feasibility of a future main trial and of delivering the stratified care intervention at the point of consultation in UK general practices.

The STarT MSK feasibility and pilot RCT (ISRCTN 15366334) was a pragmatic, two parallel arm, cluster RCT and is fully described in a separate publication [17]. In brief, 8 general practices were randomised (4 to stratified care, 4 to usual care) and patients consulting with the five most common MSK pain presentations were invited to participate in data collection over 6 months. In total, baseline questionnaires were received from 524 patients over 8 months of recruitment, with > 90% follow-up retention.

Patients seeking care within the four practices randomised to deliver stratified care received GP care guided by a bespoke stratified care management template integrated into the electronic medical record (EMR), in which the Keele STarT MSK tool and recommended matched treatment options were embedded. The template activated automatically when GPs entered one of over 200 pre-identified MSK Read-codes (i.e. symptom/ diagnostic codes) into the patient’s EMR. GPs were asked to fill-in the IT template for relevant consultations, which included completing the 9-item tool with the patient and indicating the matched treatments selected.

Delivering prognostic stratified care in MSK consultations clearly requires a change in GP and patient behaviour; not only in integrating the new tool and matched treatments as part of the IT template within consultations, but also moving away from the stepped care model often involving a predominantly biomedical approach centring on diagnosis [18], to integrating information about prognostic factors that include psychosocial obstacles to recovery. The challenges in changing consultation behaviour were highlighted in an earlier, preparatory study published in this journal, which explored patients’ and GPs’ views on the acceptability and anticipated barriers and facilitators to using stratified care [19]. Whilst this earlier work found stratified care was broadly acceptable to patients and GPs, potential barriers to its use were reported; including anticipated difficulties in integrating it within the consultation time-frame, and concerns that it may disrupt the flow of the consultation and undermine clinical autonomy. These earlier identified barriers were addressed in the subsequent design of the intervention format and content of support packages for GPs used in the feasibility and pilot trial; however, uncertainties remain as to GPs’ and patients’ willingness to engage with stratified care in the future main trial, and whether the STarT MSK tool, the IT template in which it is embedded, and matched treatments can be successfully integrated into consultations.

In addressing these uncertainties, this paper reports on findings from a nested qualitative study, which aimed to explore the feasibility of delivering the stratified care intervention ahead of the main trial. As O’Cathain et al. [20] highlight, qualitative methods are “of particular value” in exploring the feasibility of a specific intervention, through enabling “understanding of how the intervention works, and facilitate ongoing adaptation of intervention…design in preparation for a full trial”. Here we investigate GPs’ and patients’ experiences of using stratified care in consultations in order to a) understand *how* it is used, and, in particular, to b) identify barriers to its use, and c) strategies to address these. In order to do this, we draw on the COM-B model [21], discussed below. Findings were used to aid refinement of the stratified care intervention, in order to achieve strong engagement and maximise treatment fidelity in the main trial.

### The COM-B model

Using the theoretical framework of the COM-B model [21] allows us to more fully explore the aspects of patient and GP behaviour change highlighted above. The COM-B offers a way of understanding behaviour around three key determinants: *capability* – the psychological or physical ability to enact the behaviour; *opportunity* – the physical and social environment that enables the behaviour; and *motivation* – the reflective and automatic mechanisms that activate or inhibit behaviour. The COM-B model is an extension of the earlier Theoretical Domains Framework (TDF) [22], which synthesises 112 psychological constructs determining behaviour change into 14 domains, which can be used to identify barriers and facilitators to behaviour change in the context of clinical interventions. The COM-B integrates these 14 domains within its three core components. The model has been successfully used in several recent studies exploring the feasibility of delivering complex interventions [23–25]. It is particularly well-suited to exploring the feasibility of delivering stratified care, as it both provides a framework for understanding the key patient and GP barriers to the use of stratified care, as well as providing a theoretically-informed basis for the amendments subsequently made to the stratified care intervention ahead of the main trial.

## Methods

### Study design

Stimulated-recall interviews (SRIs) [26] were conducted with GPs and patients from the four stratified care intervention practices. In SRIs, video/audio recording of the interaction is used to stimulate recall in a post-consultation interview. They have been used increasingly as a way of understanding the interaction and dynamics between clinicians and patients in consultations, including in general practice [27–29]. In this study, we video- and audio-recorded consultations in which stratified care was used (*n =* 13), and then conducted separate one-to-one interviews with GPs (*n =* 10) and patients (*n =* 10) using the video/audio recording of the consultation as a prompt. Recorded consultations ranged between 9 min to 18 min in length (average 13 min). For 10 of the recorded consultations, SRIs were conducted separately with matched pairs of GPs and patients, facilitating insight into both the patient’s and GP’s individual perspectives on the same consultation. Patients involved in the other three recorded consultations were not interviewed: two declined due to lack of time and one could not be contacted; therefore, only the GPs were interviewed. These three GPs were interviewed for a second time, having already been interviewed about another recorded consultation; therefore, 13 SRIs were conducted with 10 GPs.

The study received ethical approval from the NHS REC East Midlands Nottingham 1 (Ref: 16/EM/0257).

### Patient and public involvement and engagement (PPIE)

Eight lay representatives formed a PPIE group that advised on the acceptability of methods of recruitment to the SRIs and the participant facing documentation for the nested qualitative study. Six group members also had input into data interpretation (see below).

### Recruitment and data-collection

The four intervention practices in the feasibility and pilot trial consented to take part in the qualitative study. Two of the authors (BS and BB) spent 2–3 days in each practice for recruitment purposes. At the beginning of each day recorders were set up in each GP consulting room. Patients who checked-in for an appointment were discretely approached in general practice waiting rooms − using a free-standing banner to draw attention to the study and a laminated sheet to protect patients’ privacy − to ask if they were consulting with one of the five MSK pain presentations (back, neck, shoulder, knee or multisite pain). If they were, the qualitative study was explained using a brief information sheet. Those who consented to having their consultation recorded were given a detailed information leaflet, as well as a green card to give to the GP to signal consent to record the consultation. Due to the fact that it was not possible to know in advance which patients would be consulting with one of the five MSK pain presentations on any given day, as well as the time-intensive nature of this recruitment method – which often involved researchers recruiting just one or two patients over a whole day spent in a practice ─ it was not feasible to select patients based on a purposive sampling framework to ensure a broad mix of patient characteristics. It was therefore only practically possible to sample patients on the basis of those who were consulting with one of the five MSK pain presentations of interest.

Once consenting patients had entered the consultation, if the GP judged the patient suitable ─ both for the use of stratified care and to take part in the qualitative study─ and having again checked patient consent, the recorder was turned on. GPs checked for eligibility based on i) whether the patient was in fact consulting with MSK pain rather than other painful conditions; and ii) ruling out exclusions for stratified care; for instance, a patient judged by the GP to be vulnerable (e.g. recently bereaved) or a patient requiring urgent medical attention for potentially serious and urgent pathology were ineligible. Following the consultation, the researcher gained second stage written patient consent to use the recording, and collected their telephone contact details to explore possible participation in a stimulated-recall interview (SRI). Patients who agreed were telephoned 2–3 days later, and if still willing to participate, SRI arrangements were made and a confirmation letter sent via post. Their GP was then invited via phone or email to a separate, matched SRI about the consultation.

One-to-one interviews were conducted at a single time-point with patients face-to-face (in their homes) and GPs (in their general practice) by either BS (male, PhD) or BB (female, PhD), both social science researchers with significant qualitative research experience. Patient SRIs ranged between 19 and 34 min in length (average: 22 min); GP SRIs ranged between 16 and 37 min (average: 23 min). Pre-designed topic guides were not used. Rather, prior to each interview the interviewer reviewed the recording to identify points in the consultation relevant to the delivery of the stratified care intervention. Particular parts of the consultation that were focused on when the recording was reviewed included; identifying the point at which GPs introduced the tool; understanding why GPs had chosen to use the tool at a particular point, by noting how its use was explained to patients, as well the explanation patients were given about the purpose of the tool. The use of the tool itself and the process of accessing and discussing the recommended matched treatment options were also key points of interest. For instance, the reviewer looked to identify any apparent interactional difficulties, such as if the patient experienced any difficulties in responding to any of the tool items, or if any tensions arose in the discussion of matched treatment options, in order that these could be further explored during the patients’ and GPs’ SRIs.

During SRIs, the consultation recording was used as a prompt through questions such as: “what were you thinking at that point?”, “how did you feel when asking/being asked that question?”. The intention was to explore patients’ and GPs’ recall of their experiences when using the development version of the Keele STarT MSK tool and matched treatments, and how this impacted, or facilitated, treatment decision-making. Field notes were not made during SRIs as it was felt this could negatively impact upon the flow of SRIs and the rapport between interviewer and interviewee. Written informed consent was obtained prior to these interviews, and reaffirmed verbally at the end.

### Data analysis

Interviews were audio-recorded, transcribed and anonymised. A two-stage analysis framework was adopted incorporating 1) an inductive thematic analysis, and 2) mapping identified themes onto the COM-B model components. Analysis was an iterative process and data-collection continued until sufficient saturation was judged to have been reached, defined as ‘informational redundancy’– the point at which additional data no longer offers new insights [30]. Saturation was applied principally to analysis of the interview data, rather than the consultation recordings, which were not subject to detailed coding and analysis, and as explained above were instead used as prompts in the SRIs. The number of consultation recordings was therefore only driven by saturation in so far as the number of recordings needed to achieve saturation of the SRI data.

Anonymised transcripts were first coded on a line-by-line basis by BS using Nvivo 10 software. Twenty-three initial codes were developed across the dataset as a whole, largely at a descriptive level. Codes with similar meanings were grouped together to form broader, higher level codes, which were then further abstracted, moving from description of the data to capture more conceptual interpretations. This process was guided by the constant comparison method [31], looking for connections within and across interviews, and across codes, highlighting data consistencies and variations. Whilst patient and GP data were initially coded separately, they were later mapped onto one another, looking at how the emergent findings manifested across both datasets. This again involved use of the constant comparison method to explore the similarities and variations in the properties of the codes developed from the two datasets. Whilst the GPs’ and patients’ data were found to exhibit differences in how the two groups oriented to issues relating to the use of stratified care, based on the participants’ respective roles in the consultation (as shown in the results presented later), there were broad similarities in the codes developed across the two participant groups. For instance, codes relating to GP-patient communication, the therapeutic relationship and discussion of individual treatment options were identified across both the patient and GP datasets. As a result of this similarity, it was then possible to subsume the codes into three higher-order themes to capture the breadth of the patient and GP data.

A random sample of six transcripts was independently coded by four other authors (BB, JH, JP, CCG), to explore shared meanings and interpretations, and agree the final themes. Coders brought different disciplinary perspectives to the data (BS medical sociology; BB social science; JH academic physiotherapy; JP and CCG academic general practice).

Member-checking – in terms of sending transcripts and findings to participants for comment and feedback ─ was not employed. This was principally due to the across-case, rather than within-case, focus of the analysis, which meant it would have been difficult for participants to comment on the validity of the interpretations of their own data when included within an analysis of the broader dataset. Participants were, however, given the opportunity to receive a copy of the findings upon completion of the analysis. Patient perspectives were included in the data analysis, however, as findings from the patient interview data, along with extended extracts exemplifying each theme were shared with six members of the PPIE group. A meeting was held in which the researchers looked through these data extracts with the PPIE members and explored whether their interpretations of the data aligned with the findings, and to check for alternative interpretations. All six PPIE members were broadly in agreement with the main themes identified.

Second stage analysis involved mapping the identified themes onto the COM-B model. We explored the degree to which themes ‘fitted’ within the three core components of capability, opportunity and motivation, and how the three components manifested in relation to these themes. This mapping exercise was initially carried out by BS, and then verified through detailed discussion with two other authors (BB and JH). This included examining individual data extracts exemplifying each theme, and exploring how these extracts fitted (or did not fit) within the parameters of COM-B model components. All three analysts agreed that there was a strong level of ‘fit’ between the identified themes and the three core components of the COM-B model, as outlined later in the Discussion. In what follows we detail the characteristics of the participant sample, before reporting the main themes.

## Results

### Participant characteristics

Five patients were female and five male, aged between 38 and 85 years (mean age 47), four with back pain, two neck, two knee, one shoulder, and one with multisite pain. At the point-of-consultation, four patients were stratified using the development version of the Keele STarT MSK tool at low risk, four at medium risk, and two at high risk of persistent pain. When asked in interviews, patients reported varying pain durations. Three patients reported experiencing short-term pain that had begun only a matter of weeks prior to their consultation; four reported more chronic pain problems lasting between three and 8 months; and three patients reported having had episodic pain lasting for over 20 years.

Five GPs were female and five male, and had been in practice between four and 20+ years (average 10 years approx.). As mentioned above, three of the GPs were interviewed twice about separate patient consultations. Six consultations were video-recorded and seven audio-recorded. Whilst at the start of data-collection consultations were only video-recorded, the reluctance of some patients to be videoed, along with technical difficulties experienced in switching on and off the video-recorders, led us to use the audio-recording option instead part way through data-collection.

### Main themes

The three themes identified were:

1. Integrating the Keele STarT MSK tool and IT template within consultations
2. Acceptability and suitability of tool items
3. Use of matched treatment options in guiding decision-making

#### Integrating the Keele STarT MSK tool and IT template within consultations

All GPs described initial difficulties in integrating stratified care into their patient consultations. This was partly due to challenges in completing the tool and deciding the matched treatment options within the typical 10-min consultation timeframe alongside other tasks such as defining the patient’s problem(s) and examination:

*It is a little bit clunky, there’s no two ways about it. I was interested to see how long the (video-recorded) consultations were. I noticed they were 13 min and that’s a long consultation for me; I’m usually 9–10 min and wrap up. (Female GP 1).*

The additional length added to some consultations by the tool, in particular, was also acknowledged by patients; however, some patients felt that consultations longer than the standard 10-min timeframe were in keeping with the usual ‘thorough’ approach of certain GPs:

*It went on a bit longer, probably because of that [*i.e. *the use of the tool]...but if you see him, he is always thorough, he doesn’t rush you. It’s quite normal for him.*

*(Male patient, aged 54).*

Despite GPs and some patients highlighting the time the tool added to consultations, most of the GPs also reported that having become more familiar with the tool items and scoring within the IT template, the process became faster and more streamlined:

*Now that I’m much more familiar with using it, I think I can get through the questions better. To begin with, sometimes I was hitting the wrong box or getting the ones in the wrong spaces, but now it’s fine. (Female GP 2).*

There was variation observed amongst patients in that some in fact perceived the purpose of the tool as being to help GPs to move more quickly through consultations, which was seen as being reflective of broader pressures in the healthcare system:

*I think generally doctors have got to do that now [*i.e. *use decision-aid tools], they’ve got to cut to the quick…that is just the state of the NHS [UK National Health Service] isn’t it…[the tool] has obviously been put together with a view to being helpful.*

*(Female patient, aged 71).*

Some GPs felt that the tool distracted them from the identification of potentially serious and urgent ‘red flag’ symptoms, and also was of lesser value given its focus on prognostic indicators rather than diagnosis:

*Trying to work out a diagnosis is fundamentally what you are doing. Whenever you get somebody who comes along with pain anywhere in the body, you are working out what’s underneath that pain…the vague sort of questions on the template don’t really help with formulating that diagnosis. (Male GP 1).*

There was some variation in views, however, as other GPs reported that the use of stratified care had shifted their management of patients with MSK problems, from thinking diagnostically to taking a more functional approach, taking the onus away from the GP to find a ‘solution’ to the patient’s pain problem:

*I feel very strongly that we are trained in a biomedical diagnostic model in general practice…whereas in musculoskeletal problems we are probably better off moving towards a functioning model, which [stratified care] is very much pushing us to do. It encourages people to not think there must be answer and therefore a solution, a one-off thing that a doctor can do. (Female GP 1).*

GPs suggested that the tool could sometimes interrupt the flow of the consultation and they reported having to adapt their consultation style to fit around its use:

*Because if I use the tool first and then go on the [patient] history, it doesn’t really help me…you are fitting the tool then around what’s happening really, and sadly, it’s just got in the way of the consultation. (Male GP 4).*

Some patients felt that the closed question format of the tool’s items restricted them from being able to open up discussion with the GP:

*I would have probably liked to have spoken back more but I didn’t, I just said yes or no. I would have liked to have taken part in a conversation...when I’m speaking to [the doctor] normally without this questionnaire, I have more of an input.*

*(Male patient, aged 61)*

However, other patients perceived clinical tools like the Keele STarT MSK tool as being a routine part of consultations. Rather than seeing it as presenting a barrier to pursuing key consultation goals, they saw potential added value in its use:

*It’s not surprising they use IT as a tool because you can set all the questions up, can’t you? To me it seemed quite normal, not something I wouldn’t expect or worry about… It’s probably better actually because it’s a structured approach.*

*(Male patient, aged 65).*

#### Acceptability and suitability of tool items

Five of the nine items included in the development version of the Keele STarT MSK tool were generally seen as acceptable and useful by both GPs and patients. Patients highlighted, in particular, the importance of GPs asking about psychological concerns when patients report severe pain:

*Any amount of pain that it’s so severe that you feel it’s 10/10, is going to affect you and is going to make you worry, so those are good questions...the doctor probably needs to know it’s causing anxiety. (Female patient, aged 38).*

GPs felt the tool items facilitated the opportunity for patients to raise psychological issues that may be linked to their pain, but which they may otherwise have been reluctant to mention:

*I think the tool naturally gives the patient more focus and almost permission to admit that there have been things they’ve been stewing over. (Female GP 5).*

Whist GPs reported finding the tool useful in informing their understanding of the patient’s pain, all of the GPs highlighted certain items that they felt did not work as well in the consultation as others; for instance, cases where the wording appeared awkward and did not fit within the natural flow of the conversation. The first of these was: “Do you have any other important health problems?”. GPs reported that patients often replied as though they felt the GP should already know this, or responded by simply listing all of their health conditions without indicating which they considered important:

*‘Have you got any other important health problems?’ That’s sometimes tricky because the patient has mentioned a list of things, but that qualitative measure of importance is quite tricky. The patient doesn’t necessarily say which are important, but they just produce a list. And then you’re left qualitatively deciding whether or not you think the patient thinks that’s important. (Male GP 3).*

This was also reflected in patients’ views on the same tool item, however they also reported finding it acceptable as they trusted their GP to only ask them things that were relevant:

*I did wonder why he was asking those again because he does know me very well. He knows all the problems I’ve got with my health…for many years. But I knew they would be relevant because I know he’s a good doctor. (Female patient, aged 58).*

Another tool item highlighted by GPs as problematic was: “In the last 2 weeks, have you stopped enjoying all the things you usually enjoy?”. They reported that patients often responded to this as a functional question, i.e. whether they are physically capable of doing the things they enjoy, rather than one about their mood. This perception was reflected in how patients discussed their views about this item, as in interviews they oriented to this question in terms of function:

*It’s bound to affect what you can do isn’t it…I’ve been fairly active but I’m 85 so you can’t expect a lot can you really…I find even going shopping you can’t go without a stick (Female patient, aged 85).*

*Yeah of course it [the pain] stops you doing things, of course. I particularly noticed it with the dogs because I do try to walk them but I can’t always.*

*(Female patient, aged 71).*

The item asking patients: “Do you think your pain condition will last a long time?” was also highlighted as problematic because patients often responded with “I hope not”:

*I would say 70% of people say ‘I hope not’ for that one. That leaves you wondering whether that’s a ‘yes’ or a ‘no’. I think that’s probably my most challenging question…because I think ‘I hope not’ is possibly a positive and so I ask whether that means ‘yes’? (Female GP 2).*

Finally, the item: “Do you feel it is unsafe for a person with a condition like yours to be physically active?” was identified as not working well, because GPs were concerned that this puts the idea of safety in the patient’s mind, and could have a nocebo effect:

*‘Do you think it’s unsafe?’ sometimes the patient will non-verbally jolt at that one. From our side, I guess we might worry that we’ve introduced the idea that it might be unsafe. That’s one that does occasionally feel clunky. (Male GP 5).*

#### Use of matched treatment options in guiding decision-making

Many GPs reported that they felt the recommended matched treatment options were useful generally in informing clinical management; either as a check-list to confirm they had considered all suitable management options, or in some cases providing suggestions that they may have otherwise overlooked:

*So a lot of the time it’s just confirming you’ve been through all the options that are available… it’s an aide memoir. Other times you’re kind of, ‘Actually, no, hadn’t thought of that treatment option’. (Male GP 2).*

However, in most of the recorded consultations, the use of the matched treatments was not explained to patients, and this was reflected in SRIs with patients, who generally reported being unaware as to how their answers to the tool items specifically informed subsequent treatment decisions. However, most did feel that their responses to tool items could have value in aiding the GP’s decisions, despite not being explicitly aware that their responses were informing the GP about which treatments to recommend:

*I definitely felt the approach was more thorough [when compared to previous consultations], I think it’s an additional extra…it’s good if it helps the doctor to make the correct decision. (Female patient, aged 42).*

Several patients reported that they did not feel it necessary to know explicitly how the tool informed the GP’s decision-making, and were happy to rely on the GP’s judgement in recommending treatment options:

*You trust your doctor and they’re using the questions to try and find out some degree of how bad your back is and then to decide what action we need to take.*

*(Male patient, aged 44).*

In some SRIs, GPs reported that they had already made up their mind about treatment prior to completing the tool; they therefore completed the stratified care template mainly because they were participating in the pilot RCT, rather than using the tool to guide their decision-making about which matched treatment option(s) to recommend:

*Would it change what I do? I don’t think it probably will because I’ve usually decided what I’m going to do with them. [In the recorded consultation] I knew the tool wasn’t probably going to change anything that I did, it was sort of, ‘oh now I’ve got to fill this in’ [the Keele STarT MSK template] (Female GP 1).*

In a few of the consultations, GPs opted to refer the patient for an MRI scan (which, given this is an imaging request rather than a treatment option, was not included in the recommended treatment options) and not to select one of the recommended matched options. Referring patients for a scan did not necessarily indicate GPs acting against the recommendations of the tool – as the prognostic stratification was intended to supplement, not replace, a diagnostic approach; nonetheless, some GPs highlighted that in certain instances they saw a scan as being more appropriate than the recommended treatment options:

*I guess it wouldn’t always feel terribly comfortable for this type of patient to opt down an immediate management pathway rather than a diagnostic pathway. Not least when she’s already said that she’s worried about something sinister going on. I felt duty bound to deal with that anxiety. She’s expressly asked about imaging, so I felt…it was probably better to image her. (Male GP 3).*

In relation to the patient discussed in this extract, the matched SRI with the patient highlighted her sense of reassurance and satisfaction in being referred for an MRI scan following discussion with the GP:

*This is a different kind of pain that I’ve got at the moment. It’s much more severe. I’ve [previously] had an x-ray on my hips and my knees and my spine. But he’s sending me for an MRI scan, he explained the difference to me. That will be even more helpful, it’s going to be better. Yes, I’m happy with that. I felt relieved and reassured. (Female patient, aged 61).*

Some matched treatment options, e.g. referral to pain management clinics, were identified by both patients and GPs as being of lesser value given the difficulty of accessing such services either because they were unavailable in the local area or because of long NHS waiting lists:

*If you’ve got a bad back it’s annoying and it drags you down, it’s painful and you can’t walk properly but you’ve got to wait months and months [for an appointment at the pain services]; it’s a long time to wait. (Male patient, aged 51).*

*There is the pain management clinic referral on there, isn’t there? I can’t remember really the last time I’ve used that simply because the waiting times are so horrifically long. (Female GP 4).*

## Discussion

This study explored the feasibility of delivery of stratified primary care for patients with musculoskeletal pain, within the context of the STarT MSK feasibility and pilot cluster RCT. Key uncertainties we looked to address included GPs’ and patients’ willingness to engage with stratified care in the future main trial, and whether the Keele STarT MSK tool, IT template, and matched treatment options could be successfully integrated into consultations.

Findings indicate that delivery of the stratified care intervention is in some respects feasible; as both GPs and patients saw added value in some of the tool questions; and GPs often found the matched treatment options useful, either in guiding or confirming their management decisions. Findings also suggest, however, that aspects of the intervention required amending before progressing to the main trial. In the case of some tool items, GPs reported difficulty in communicating to patients the underlying constructs. Additionally, some matched treatment options were perceived to be less useful because of the lack of availability, or difficulty accessing them; whilst other treatment options were highlighted that were seen as suitable for some patients, but which were not currently included in the recommended matched treatments. GPs also reported difficulty integrating the tool alongside other clinical tasks; although many indicated that this became easier the more they used it. As outlined in the Methods, earlier, the GPs interviewed had a wide range of experience levels (between four and 20+ years). Given that similar views were reported amongst GPs across the data about integrating the tool in consultations, the length of time in practice of the GP did not appear to have an impact on the degree of ease or difficulty experienced in this regard.

To more fully understand the feasibility of delivery of stratified care in a future main trial we will now discuss these findings through the lens of the three core components in the COM-B model.

### Capability

Both patients and GPs identified a number of ‘cumbersome’ tool items which made the STarT MSK tool awkward to use and make sense of at an individual patient level, often posing difficulties for patients in understanding the concept behind the tool’s items, and for GPs in interpreting patients’ responses. This affected the *capability* of GPs and patients to effectively engage with the stratified care approach.

### Opportunity

A tension was present in that GPs and patients felt the tool facilitated the opportunity to introduce psychological concerns that may otherwise not have been raised, yet patients also highlighted that the closed nature of the tool items restricted their opportunity to engage in a discussion about these issues. GPs identified as a barrier the added time taken in a typical 10-min consultation, a continuing challenge identified in our earlier work, as well as that of others [32]. This reflects the environmental context of primary care consultations in the UK, and is not modifiable within the constraints of a pragmatic trial. Likewise, the lack of availability of certain recommended matched treatment options, e.g. pain management clinics, and long waiting times for some services indicates a clear lack of *opportunity* for both GPs and patients to fully utilise the stratified care approach due to barriers at a broader NHS service availability level.

### Motivation

GPs appeared to have greater motivation to use the STarT MSK tool as a decision-aid if they perceived it as having added value to the individual patient. Those who favoured a diagnostic approach were less inclined to use it and only did so because they were participating in the feasibility and pilot trial. Motivation appeared less relevant for patients, given that most were unaware that decisions about their treatment were linked to their responses to the tool item questions. A more relevant construct for patients appeared to be trust − having trust in the GP’s knowledge and expertise was key to their views of treatment decisions.

Mapping findings onto the COM-B model enabled targeting of specific behaviour change determinants through amendments to the stratified care intervention ahead of the main trial, as will be detailed further below.

Findings reported here show both similarities and differences with other qualitative studies into stratified care for MSK pain [32–34], including our own earlier paper [19]. In our 2016 paper, some similar barriers were anticipated to those found here, such as difficulty integrating the tool within the consultation time-frame; disruption to the flow of the consultation; and the lack of availability of some matched treatment options. However, the present findings also indicate a more positive outlook, in that GPs reported being able to integrate stratified care more easily within the consultation as they became more familiar with the tool and matched treatments. Additionally, whilst in our earlier paper both GPs and patients expressed concerns that the use of the tool could undermine clinical autonomy, reflecting similar findings in the broader health services literature vis-à-vis concerns about the use of clinical tools [35, 36], this concern did not emerge here. On the contrary, patients reported added value in that stratified care enabled a more ‘structured’ consultation, and in some instances GPs reported selecting treatment options based on their clinical judgement rather than being guided by the matched treatment recommendations. The difference between the two studies may reflect the degree to which we were able to successfully address these earlier identified barriers in our design of the stratified care intervention and the training and support for participating GPs.

Previous research exploring GPs’ views of stratified care for low back pain (LBP) in the UK found that GPs did not ‘buy in’ to the use of stratified care, and used the approach only through a sense of obligation due to it being part of a research study [34]. There was some evidence of this in our findings as well; however, GPs and patients also emphasised the added value they saw in the approach, and some GPs reported that using stratified care had resulted in a positive shift in their management of MSK conditions, away from focusing on diagnosis towards thinking about function.

In exploring GPs’ views on stratified care for LBP in Germany, Karstens et al. [33] reported the potential for stratified care to negatively impact upon the therapeutic relationship through undermining GP-patient rapport, a similar finding in our 2016 paper. This partly supports our present finding that patients perceived the closed nature of tool items as hindering the discussion of certain issues, which may indicate some negative impacts on GP-patient communication. However, there was variation in patients’ views in our study, as several patients in fact felt that the tool items enabled them to voice worries and concerns to the GP. This variation suggests that whilst for some patients the use of the Keele STarT MSK tool represented a barrier to productive communication, conversely, for others it was a facilitator. The latter finding supports Hsu et al.’s [32] findings that primary care clinicians in the US reported that using the STarT Back tool facilitated them in opening up conversations with patients about their concerns; however, their study did not include patients’ perspectives.

#### Amendments to the stratified care intervention informed by the qualitative findings

Amendments to the tool and matched treatment options were informed by bringing together the qualitative findings reported here with the quantitative findings from the feasibility and pilot trial, which are reported elsewhere (see Hill et al. [17]). The changes, as outlined below, were made following a series of discussions amongst the broader TAPS trial team, as well incorporating input from members of the TAPS Trial Steering Committee and the study’s PPIE group.

In order to increase GPs’ and patients’ capability to effectively use the tool, a new clinical version of the tool was developed (to supplement the self-report version of the tool), which would be more intuitive for GPs to ask during the consultation. Four items in the development version of the tool were adapted to more clearly capture the underlying construct, e.g. to better distinguish between mood and function. Headings were also added to each of the tool items to remind GPs of the underpinning constructs in order to help them effectively communicate these to patients.

To increase both opportunity and motivation to engage with the matched treatment options, these were refined in ways that reflected the views and experience of participating GPs, e.g. ‘consider imaging’ and ‘GP management of comorbidities, distress, frailty, polypharmacy and pain management’.

The new clinical version of the Keele STarT MSK tool and revised matched treatment options are currently being tested as part of a main trial investigating the clinical and cost-effectiveness of stratified primary care versus usual, non-stratified care for patients presenting with one of the five most common MSK pain presentations in primary care.

With regard to maximising opportunity to use stratified care in the main trial, findings also highlighted the need to closely support GPs in the main trial to effectively integrate the tool and matched treatment options in consultations; our approach to this will be detailed in a separate forthcoming paper.

#### Strengths and limitations

A strength of this study is the SRI method, as the use of the consultation recording enhanced the interviews, minimising recall bias. The use of the COM-B model is also a strength in that it enabled us to develop a theoretically-informed understanding of the key behaviour change areas to address in order to maximise engagement with stratified care in the main trial. The multidisciplinary team involved in data analysis was a further strength; as well as PPIE input into the interpretation of the patient data, which increases the trustworthiness of the findings presented.

A potential limitation is the possibility of an observer’s paradox effect [37] where, as a result of being recorded, GPs may have altered their consulting style, or engaged with the tool and matched treatments differently to how they would have done ordinarily. Additionally, the decision part way through data-collection to opt for audio-recording consultations rather than video-recording meant that we lost some of the richness of the subsequent data in terms of being able to see visually how stratified care was used within the consultation. This change was necessary, however, in order to achieve sufficient saturation within the recruitment timeframe of the feasibility and pilot trial.

When interpreting these findings, it is also important to acknowledge the influence of the researchers’ contributions on participants’ responses in SRIs. The two interviewers were part of the wider STarT MSK programme team, and therefore their close involvement with the pilot RCT could have had the potential to influence the way in which participants’ views were elicited in SRIs. It was made explicit to participants prior to SRIs that the interviewers were part of the study team that was developing and testing the stratified care intervention; however, it was also emphasised to participants that the aims of qualitative study were exploratory and therefore the interviewers were interested in investigating both positive and negative aspects of participants’ experiences. As such, the researchers made a conscious effort not to impose their own priorities on the data collection, and the variation in views observed suggests that participants were not led into adopting a particular stance.

## Conclusion

Through the use of matched stimulated-recall interviews aided by consultation recordings, analysed using the COM-B model, we were able to gain an in-depth, theoretically-informed understanding of the feasibility of delivery of stratified care in a future main trial. Findings indicate that both GPs and patients saw added value in the use of stratified care in the pilot and feasibility trial, but barriers to its use were also identified in relation to the capability, opportunity and motivation the two participant groups felt they had to engage with it. To bring about behaviour change in terms of GPs and patients fully engaging with the Keele STarT MSK tool and matched treatment options in the future main trial, the tool items must be clearly worded, easily comprehensible and consistent with the conversational style of the consultation; matched treatment options need to be readily available and seen as having added clinical value; and the overall approach must integrate well with other elements of the consultation. Findings have informed key changes to the stratified care intervention currently being tested in the main STarT MSK trial, and can also have implications for informing the design of other similar complex interventions in primary care settings.

## Data Availability

In line with the Standard Operating Procedures in place at the School of Primary, Community and Social Care, where this study was conducted, data are archived at a dedicated location within the Keele University’s network. A request to access archived data can be made by completion of a Data Transfer Request form, which can be accessed by contacting: Primary Care Centre Versus Arthritis, School for Primary, Community and Social Care, Keele University, Staffordshire, ST5 5BG, UK; Tel: + 44 (0) 1782 733905.

## Abbreviations

COM-B: Capability, Opportunity, Motivation and Behaviour
GP: General Practitioner/ General Practice
RCT: Randomised Controlled Trial
